# T-type calcium channel enhancer SAK3 promotes dopamine and serotonin releases in the hippocampus in naive and amyloid precursor protein knock-in mice

**DOI:** 10.1371/journal.pone.0206986

**Published:** 2018-12-20

**Authors:** Shuo Wang, Yasushi Yabuki, Kazuya Matsuo, Jing Xu, Hisanao Izumi, Kenji Sakimura, Takashi Saito, Takaomi C. Saido, Kohji Fukunaga

**Affiliations:** 1 Department of Pharmacology, Graduate School of Pharmaceutical Sciences, Tohoku University, Sendai, Japan; 2 Department of Cellular Neurobiology, Brain Research Institute, Niigata University, Niigata, Japan; 3 Laboratory for Proteolytic Neuroscience, RIKEN Brain Science Institute, Wako-shi, Saitama, Japan; University Paris Diderot, FRANCE

## Abstract

T-type calcium channels in the brain mediate the pathophysiology of epilepsy, pain, and sleep. Recently, we developed a novel therapeutic candidate, SAK3 (ethyl 8'-methyl-2',4-dioxo-2-(piperidin-1-yl)-2'H-spiro[cyclopentane-1,3'-imidazo[1,2-a] pyridine]-2-ene-3-carboxylate), for Alzheimer’s disease (AD). The cognitive improvement by SAK3 is closely associated with enhanced acetylcholine (ACh) release in the hippocampus. Since monoamines such as dopamine (DA), noradrenaline (NA), and serotonin (5-HT) are also involved in hippocampus-dependent learning and psychomotor behaviors in mice, we investigated the effects of SAK3 on these monoamine releases in the mouse brain. Oral administration of SAK3 (0.5 mg/kg, p.o.) significantly promoted DA and 5-HT releases in the naive mouse hippocampal CA1 region but not in the medial prefrontal cortex (mPFC), while SAK3 did not affect NA release in either brain region. The T-type calcium channel-specific inhibitor, NNC 55–0396 (1 μM) significantly antagonized SAK3-enhanced DA and 5-HT releases in the hippocampus. Interestingly, the α7 nicotinic ACh receptor (nAChR) antagonist, methyllycaconitine (1 nM) significantly inhibited DA release, and the α4 nAChR antagonist, dihydro-β-erythroidine (100 μM) significantly blocked both DA and 5-HT releases following SAK3 (0.5 mg/kg, p.o.) administration in the hippocampus. SAK3 did not alter basal monoamine contents both in the mPFC and hippocampus. SAK3 (0.5 mg/kg, p.o.) administration also significantly elevated DA and 5-HT releases in the hippocampal CA1 region of amyloid-precursor protein (APP)^NL-GF^ knock-in (KI) mice. Moreover, hippocampal DA and 5-HT contents were significantly decreased in APP^NL-GF^ KI mice. Taken together, our data suggest that SAK3 promotes monoamine DA and 5-HT releases by enhancing the T-type calcium channel and nAChR in the mouse hippocampus.

## Introduction

Monoamines including dopamine (DA), serotonin (5-HT), and noradrenaline (NA) mediate various central nerve system functions such as motivation, motor function, and cognition [1,2]. Dysregulation of monoamine systems is associated with various psychiatric and neurodegenerative disorders [3]. In patients with schizophrenia, mesocorticolimbic DA dysfunction accounts for both psychotic and cognitive disturbances. Anti-psychotics with DA receptor blockers, such as risperidone, are generally used for therapy [4,5]. In addition, blockade of 5-HT and NA reuptake is the most common target of therapeutics for depression and behavioral and psychological symptoms of dementia (BPSD) in patients with Alzheimer’s disease (AD) [6,7]. Furthermore, 5-HT levels are markedly reduced in the cerebral limbic and basal ganglia areas in patients with AD compared to healthy subjects [8,9]. These reports indicated that dysregulation of monoamine levels has a critical role in psychomotor disturbance in both psychiatry diseases and AD.

T-type calcium channels, known as transient and low voltage-activated calcium channels, are characterized as electrophysiological kinetics by fast inactivation and slow deactivation [10,11]. All Cav3.1, Cav3.2, and Cav3.3 T-type calcium channels are expressed in the brain and maintain the physiological and pathological systems [12–16]. Previously, we developed the cognitive enhancer, ST101 (spiro [imidazole [1.2-a] pyridine-3, 2-indan]-2(3H)-one), which enhances Cav3.1 T-type calcium channel current in Cav3.1-transfected neuro2A cells [17]. ST101 significantly enhanced calcium/calmodulin-dependent protein kinase II and in turn promoted long-term potentiation in rat somatosensory cortical slices; these effects were blocked by the T-type calcium channel inhibitor, mibefradil [17]. We also generated a more potent T-type calcium channel enhancer, SAK3 (ethyl 8'-methyl-2',4-dioxo-2-(piperidin-1-yl)-2'H-spiro[cyclopentane-1,3'-imidazo [1,2-a] pyridine]-2-ene-3-carboxylate) [18]. SAK3 potentiates Cav3.1 and Cav3.3 currents, which display a more potent effect than ST101 [18]. Acute SAK3, but not ST101, (0.5 mg/kg, p.o., each) administration increased acetylcholine (ACh) release in the hippocampus, thereby improving memory impairments seen in olfactory bulbectomized mice [18]. Moreover, SAK3 prevents neuronal cell death in hippocampal CA1 pyramidal neurons followed by transient brain ischemia through nicotinic ACh receptor (nAChR) stimulation [18,19]. Therefore, SAK3 may activate nAChR signaling by promoting hippocampal ACh release through enhancing T-type calcium channels. However, the effects of SAK3 on monoamine release remain unclear.

In this context, we investigated the effects of SAK3 on monoamine release in the mouse medial prefrontal cortex (mPFC) and hippocampal CA1 region. We also evaluated the effects of SAK3 (0.5 mg/kg, p.o.) on monoamine release in the hippocampus in amyloid precursor protein (APP)^NL-GF^ knock-in (KI) mice as an animal model of AD [20]. Our results provide evidence that T-type calcium channel stimulation can increase monoamine release in both physiological and pathological conditions.

## Materials and methods

### Animals

Male 6-week-old ddY mice were purchased from Clea Japan, Inc. (Tokyo, Japan). APP^NL-GF^ KI mice were obtained from Dr. Takashi Saito and Dr. Takaomi C Saido (Riken, Saitama, Japan). Cav3.1 knock-out (KO) mice were generated by Dr. Kenji Sakimura [21]. Wild-type (WT) C57BL/6J mice were also purchased from Clea Japan, Inc. (Tokyo, Japan). Animals were housed under conditions of constant temperature (23 ± 2°C) and humidity (55 ± 5%) on a 12-h light-dark cycle (light from 9 am–9 pm) and fed with standard forage. Animals were euthanized by isoflurane overdose or cervical dislocation after experiments. All animal procedures were approved by the Committee on Animal Experiments of Tohoku University.

### Reagents

**Fig 1:**
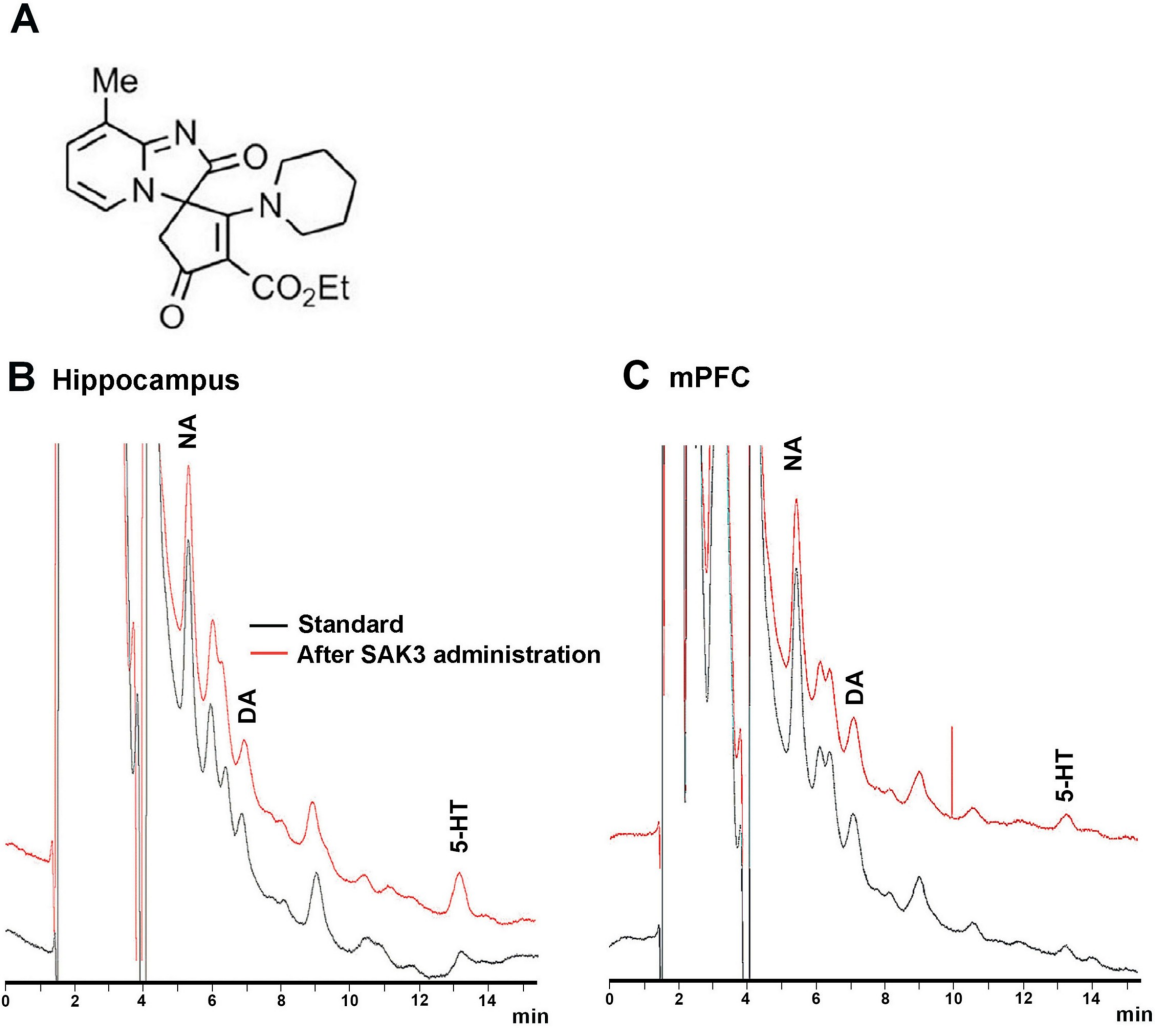
Representative patterns of chromatogram of NA, DA, and 5-HT. (A) The structure of SAK3. (B) Peak patterns of NA, DA, and 5-HT in dialysate from the hippocampus. (C) Peak patterns of NA, DA and 5-HT in the mPFC. NA, DA, and 5-HT peaks appear around 5, 7, and 13 min, respectively. NA: noradrenaline, DA: dopamine, 5-HT: serotonin, mPFC: medial prefrontal cortex.

SAK3 was synthesized by Shiratori pharmaceutical Ltd (Chiba, Japan; Fig 1A) according to a previous study [18]. As SAK3 (0.5 mg/kg, p.o.) shows maximal effects of ACh release and significant cognitive enhancement in several animal models including APP^NL-F^ KI mice [18,19,22], we chose dose of SAK3 at 0.5 mg/kg to evaluate monoamine release. SAK3 was dissolved in distilled water. T-type calcium channel inhibitor, NNC 55–0396 (1 μM: Sigma-Aldrich, St-Louis, MO) [23], TTA-A2 (1 μM: Alomone Labs, Jerusalem, Israel), α7 nAChR antagonist MLA (1 nM: Sigma-Aldrich), and α4β2 nAChR antagonist DhβE (100 μM: Tocris, Bristol, UK) were dissolved in Ringer’s solution.

### Measurement of monoamine releases using *in vivo* microdialysis

Stereotaxic surgery for *in vivo* microdialysis in mice was performed as previously described [24]. Mice were anesthetized with pentobarbital Na (50 mg/kg, i.p.), and the head was placed in a stereotaxic apparatus (David Kopf Instruments, Tujunga, CA, U.S.A.). A guide cannula (AG-4 for the hippocampus or AG-3 for the mPFC; Eicom, Kyoto, Japan) was inserted into the dorsal hippocampal CA1 region [2.9 mm posterior and 3.3 mm lateral to the bregma and 1.4 mm below the brain surface, according to [25]] or the mPFC [1.9 mm posterior and 0.3 mm lateral to the bregma and 1.8 mm below the brain surface, according to [25]], and the skull was covered by dental cement. The microdialysis probe (A-I-4-02 for the hippocampus or A-I-3-02 for the mPFC; Eicom) was inserted through the guide cannula. After recovery, Ringer’s solution was perfused at 2 μL/min using a micro-syringe pump (ESP-64; Eicom) under free moving conditions. PP-ODS (Eicom) was used for measurement of hippocampal DA and 5-HT [24]. Hippocampal NA and monoamines in the mPFC were measured using CAX-EICOMPAK (Eicom). Perfused dialysates were collected every 6 min (PP-ODS) or 15 min (CAX-EICOMPAK) in the sample loop of an auto-injector (EAS-20; Eicom) connected to a high-performance liquid chromatography (HPLC)-electrochemical detector (ECD) system (HTEC-500; Eicom). When monoamine levels reached a steady state, mice were treated with SAK3 (0.5 mg/kg, p.o.). The T-type calcium channel specific blocker, NNC 55–0396 (1 μM), the α7 nAChR antagonist, MLA (1 nM), or the α4β2 nAChR antagonist, DhβE (100 μM) in Ringer’s solution was infused to brain regions through a microdialysis probe before SAK3 administration. Monoamine levels were calculated in the chromatogram (Fig 1B and 1C). Monoamine release was assessed as a percentage of basal levels. Released monoamine levels were calculated after SAK3 treatment by comparison to the responses of vehicle-treated animals at the same time points.

### Measurement of monoamine contents in the brain tissues

Fifteen minutes after SAK3 (0.5 mg/kg, p.o.) administration, monoamine contents were measured in ddY mice. Ten-month-old WT and APP^NL-GF^ KI mice were used for the measurement. Animals were sacrificed by cervical dislocation for dissection of brain tissues. After decapitation, the mPFC and hippocampal CA1 region were dissected, frozen in liquid nitrogen, and stored at -80°C until assayed. Analyses of monoamine contents were performed as previously described [24]. Each frozen tissue sample was weighed and homogenized in 200 μL of 0.2 M perchloric acid containing 100 ng/mL isoproterenol as an internal standard. The homogenate was placed on ice for 30 min and then centrifuged at 20,000 x g for 15 min at 4°C. Monoamine contents in the supernatants were quantified with the HPLC-ECD system and expressed as ng/g tissue weight.

### Statistical analysis

Significant differences were determined using Student's *t*-test for two-group comparison and by two-way analysis of variance (ANOVA) for multi-group comparisons to analyze *in vivo* microdialysis. Other comparison between multiple groups was performed using one-way ANOVA followed by Tukey’s multiple comparisons test. Results are expressed as mean ± standard error of the mean (SEM).

## Results

### Acute SAK3 administration promotes DA and 5-HT releases in the hippocampal CA1 region

**Fig 2:**
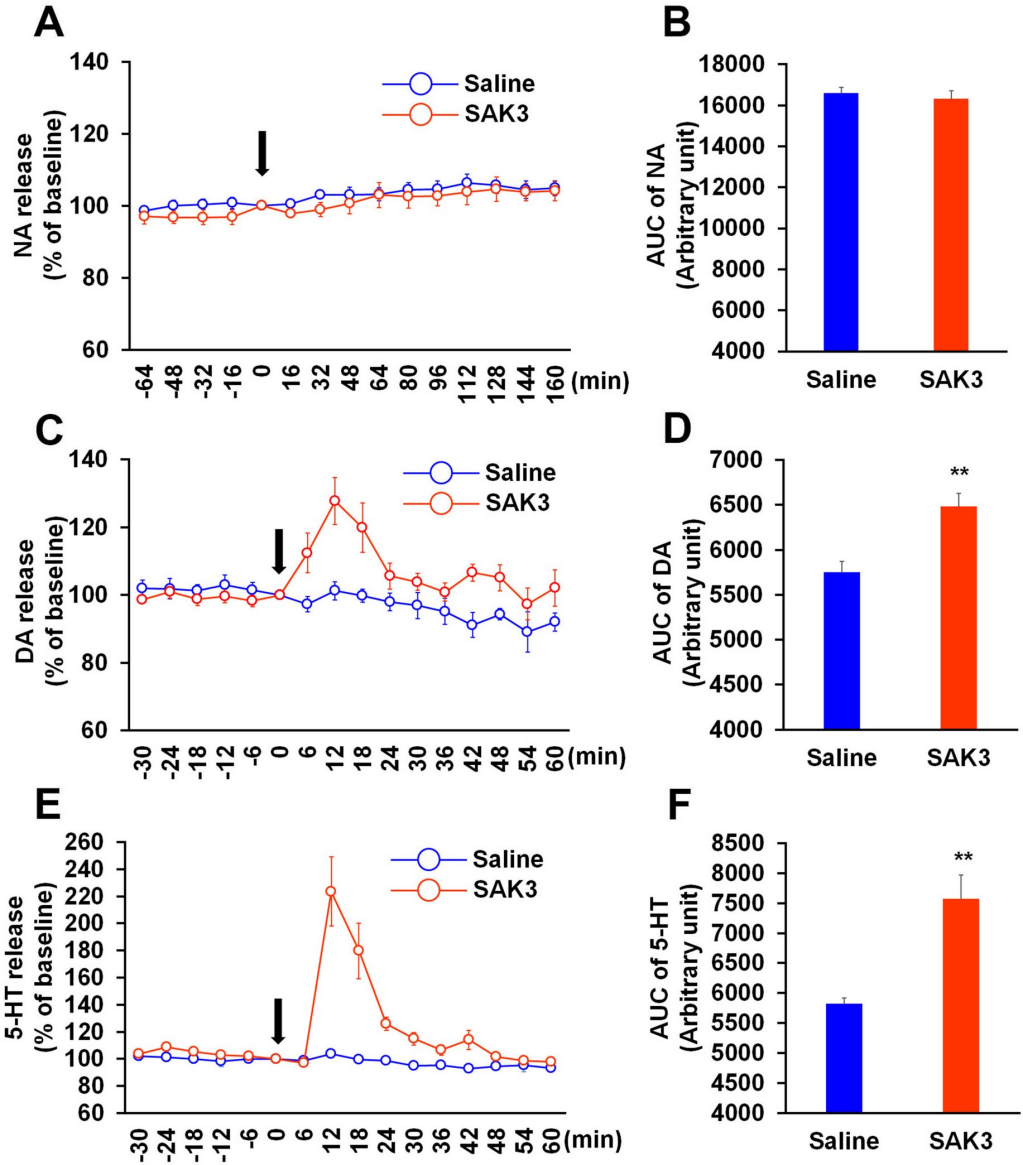
Effects of SAK3 on monoamine releases in the hippocampus. (A, C, E) Time zero defines the point immediately following SAK3 oral administration. NA levels in the dialysate were analyzed every 16 min in the hippocampal CA1 region (n = 6 per group). DA and 5-HT levels in the dialysate were analyzed every 6 min (n = 10 per group). (B, D, F) NA, DA, and 5-HT levels were calculated with the AUC. ** p < 0.01 vs. saline treated mice. Error bars represent the SEM. NA: noradrenaline, DA: dopamine, 5-HT: serotonin, AUC: area under the receiver operator characteristic curve.

We first investigated the effect of SAK3 on NA, DA, and 5-HT releases in the hippocampal CA1 region. NA, DA, and 5-HT levels were calculated by the area under the receiver operator characteristic curve (AUC: NA levels were calculated from time 0 to 160 min, DA and 5-HT levels were calculated from time 0 to 60 min). Acute SAK3 (0.5 mg/kg, p.o.) administration significantly promoted DA and 5-HT releases with a peak at 12 min in the hippocampal CA1 region (DA: p = 0.0011 vs. saline-treated mice; 5-HT: p = 0.0005 vs. saline-treated mice; Fig 2C, 2D, 2E and 2F). On the other hand, SAK3 (0.5 mg/kg, p.o.) administration did not affect NA release (p = 0.5494 vs. saline treated mice; Fig 2A and 2B). Two-way repeated measures ANOVA analysis revealed a significant effect of SAK3 administration on DA [F (1, 18) = 9.778, p = 0.0058] and 5-HT [F (1, 18) = 18.34, p = 0.0004], an effect of time [DA: F (15, 270) = 4.11, p < 0.0001; 5-HT: F (15, 270) = 4.31, p < 0.0001], and a significant time × SAK3 administration interaction [DA: F (15, 270) = 3.444, p < 0.0001; 5-HT: F (15, 270) = 3.628, p < 0.0001] (Fig 2C and 2E). In NA release, two-way repeated measures ANOVA analysis revealed a significant effect of time [F (14, 140) = 6.147, p < 0.0001], but an effect of SAK3 administration [F (1, 10) = 0.7369, p = 0.4108] and time × SAK3 administration interaction [F (14, 140) = 0.3801, p = 0.9786] were not significant (Fig 2A). We also evaluated DA and 5-HT contents of hippocampal tissues dissected 15 min after SAK3 administration. However, SAK3 (0.5 mg/kg, p.o.) administration did not alter basal DA and 5-HT contents compared to saline-treated mice (DA: p = 0.2604 vs. saline treated mice; 5-HT: p = 0.7999 vs. saline treated mice; Table 1).

**Table 1:** Monoamine contents by single administration of SAK3 (0.5 mg/kg, p.o.) administration in the mPFC and hippocampal CA1 region in naïve mice.

mPFC	Saline	SAK3
DA (ng/g)	105.1 ± 44.9	193.3 ± 145.4
5-HT (ng/g)	224.1 ± 29.7	313.9 ± 41.9
**Hippocampus**	**Saline**	**SAK3**
DA (ng/g)	10.2 ± 5.2	3.0 ± 0.9
5-HT (ng/g)	157.3 ± 18.7	170.2 ± 10.4

Acute SAK3 (0.5 mg/kg, p.o.) administration did not alter DA and 5-HT contents in both brain regions in naive mice (n = 5 per group).

mPFC: medial prefrontal cortex, DA: dopamine, 5-HT: serotonin

### Acute SAK3 administration does not affect monoamine release in the mPFC

**Fig 3:**
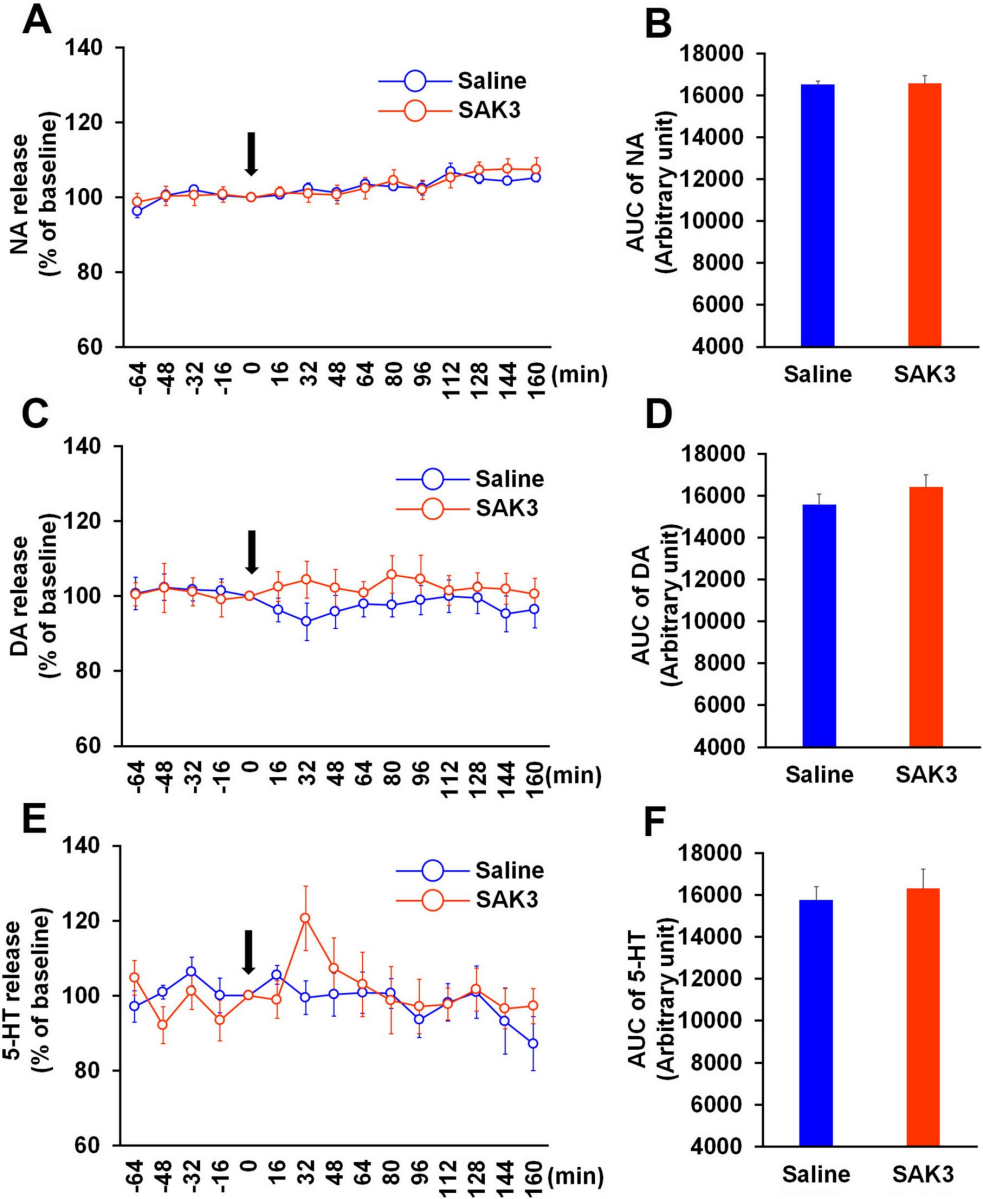
Effects of SAK3 on monoamine release in the mPFC. (A, C, E) Time zero defines the point immediately after SAK3 oral administration. NA, DA, and 5-HT levels in the dialysate were analyzed every 16 min in the mPFC (n = 9 per group). Error bars represent the SEM. (B, D, F) NA, DA, and 5-HT levels were calculated with the AUC (n = 9 per group). Error bars represent the SEM. mPFC: medial prefrontal cortex, NA: noradrenaline, DA: dopamine, 5-HT: serotonin, AUC: area under the receiver operator characteristic curve.

Since monoamine levels in the mPFC mediate cognition and psychic functions [26, 27], we next tested whether SAK3 promotes monoamine release in the mPFC. In contrast to the hippocampal CA1 region, acute SAK3 (0.5 mg/kg, p.o.) administration did not affect NA, DA, and 5-HT releases in the mPFC (NA: p = 0.8793 vs. saline-treated mice; DA: p = 0.2874 vs. saline-treated mice; 5-HT; p = 0.6314 vs. saline-treated mice; Fig 3). Two-way repeated measures ANOVA analysis revealed several significant effect on NA (SAK3 administration [F (1, 16) = 0.0298, p = 0.8651], time [F (14, 224) = 9.518, p < 0.0001] and time × SAK3 administration interaction [F (14, 224) = 0.8554, p = 0.6083]; Fig 3A) and 5-HT (SAK3 administration [F (1, 16) = 0.08784, p = 0.7708], time [F (14, 224) = 2.458, p = 0.003] and time × SAK3 administration interaction [F (14, 224) = 1.865, p = 0.0312]; Fig 3E). There were no significant effect of SAK3 administration [F (1, 16) = 0.6613, p = 0.428], time [F (14, 224) = 0.3529, p = 0.9856] and time × SAK3 administration interaction [F (14, 224) = 0.8443, p = 0.6204] on DA releases in mPFC (Fig 3C). In addition, DA and 5-HT contents in the mPFC were also unaffected by SAK3 (0.5 mg/kg, p.o.) administration (DA: p = 0.7643 vs. saline-treated mice; 5-HT; p = 0.3410 vs. saline-treated mice; Table 1).

### T-type calcium channel inhibitor and nAChR antagonists prevent SAK3-promoted DA and 5-HT releases in the hippocampal CA1 region

**Fig 4:**
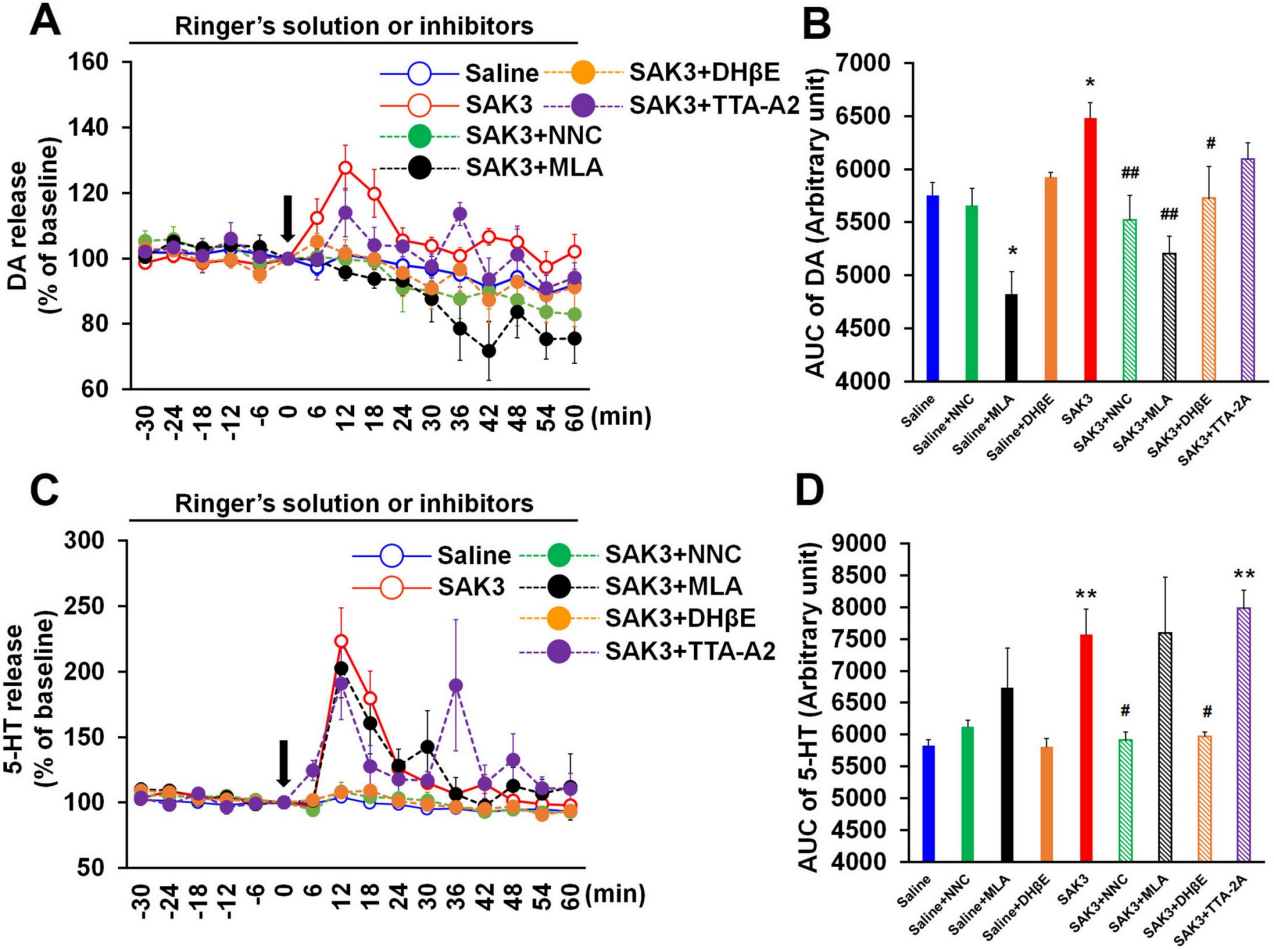
SAK3 may promote DA and 5-HT release via stimulating T-type calcium channels and nAChRs. (A) NNC 55–0396 (1 μM), TTA-A2 (1 μM), DhβE (100 μM), or MLA (1 nM) treatment through the microdialysis probe inhibited DA release following SAK3 (0.5 mg/kg, p.o.) administration in the CA1 region (n = 4–10 per group). Error bars represent the SEM. (B) AUC of DA levels at time point from 0 to 60 min were calculated. *p < 0.05 vs. saline-treated mice; #p < 0.05 vs. SAK3-treated mice; ##p < 0.01 vs SAK3-treated mice. Error bars represent the SEM. (C) NNC 55–0396 (1 μM) or DhβE (100 μM) treatment through the microdialysis probe antagonized 5-HT release by SAK3 (0.5 mg/kg, p.o.) administration in the CA1 region (n = 4–10 per group). Error bars represent the SEM. (D) AUC of 5-HT levels at time point from 0 to 60 min were calculated. **p < 0.01 vs. saline-treated mice; #p < 0.05 vs. SAK3-treated mice. Error bars represent the SEM. nAChR: nicotinic acetylcholine receptor, DA: dopamine, 5-HT: serotonin, AUC: area under the receiver operator characteristic curve.

**Fig 5:**
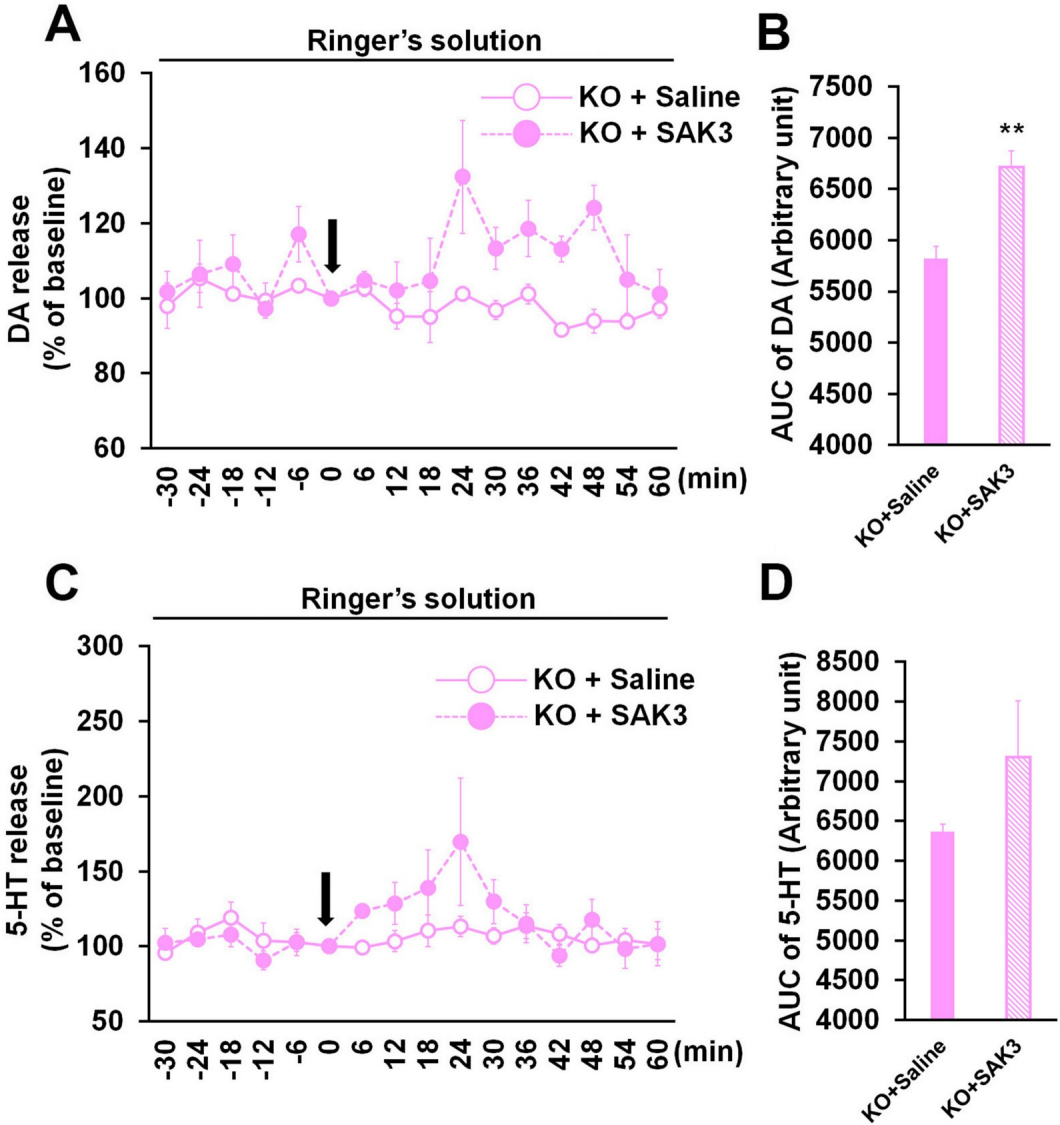
Effect of SAK3 on DA and 5-HT releases in the Cav3.1 KO hippocampus. (A, C) Time zero defines the point immediately after saline or SAK3 oral administration. DA (A) and 5-HT (C) levels in the dialysate were analyzed every 6 min in the hippocampus in Cav3.1 KO mice (n = 4 per group). Error bars represent the SEM. (B, D) DA (B) and 5-HT (D) levels at time point from 0 to 60 min were calculated with the AUC (n = 4 per group). Error bars represent the SEM. **p < 0.01 vs. saline-treated Cav3.1 KO mice. Error bars represent the SEM. DA: dopamine, 5-HT: serotonin, AUC: area under the receiver operator characteristic curve, KO: Cav3.1 KO mice.

We previously reported that SAK3 promotes ACh release through enhancing T-type calcium channels in the mouse hippocampus [18]. In addition, SAK3 shows neuroprotective effects against transient brain ischemia via activation of nAChR in the CA1 pyramidal neurons [19]. Thus, we tested whether the T-type calcium channel inhibitor or nAChR antagonists eliminate SAK3-promoted DA and 5-HT releases in the mouse hippocampus. Significant group effects were observed in DA [F (8, 48) = 7.384, p < 0.0001] and 5-HT [F (8, 48) = 6.452, p < 0.0001] levels (Fig 4B and 4D). T-type calcium channel specific inhibitor NNC 55–0396 (1 μM) significantly antagonized SAK3-enhanced DA and 5-HT releases in the hippocampal CA1 region (DA: p = 0.9805 vs. saline-treated mice, p = 0.0067 vs. SAK3-treated mice; 5-HT: p > 0.9999 vs. saline-treated mice, p = 0.0383 vs. SAK3-treated mice; Fig 4B and 4D). On the other hand, other T-type calcium channel inhibitor TTA-A2 (1 μM) partially blocked SAK3-enhanced DA but not 5-HT releases (DA: p = 0.3478 vs. saline-treated mice, p = 0.2346 vs. SAK3-treated mice; 5-HT: p < 0.0001 vs. saline-treated mice, p = 0.2409 vs. SAK3-treated mice; Fig 4B and 4D). Both α4β2 nAChRs antagonist, dihydro-β-erythroidine (DhβE: 100 μM) and α7 nAChR antagonist, methyllycaconitine (MLA: 1 nM) significantly inhibited DA release by SAK3 (0.5 mg/kg, p.o.) administration (DHβE: p > 0.9999 vs. saline-treated mice, p = 0.0388 vs. SAK3-treated mice; MLA: p = 0.3448 vs. saline-treated mice, p = 0.0001 vs. SAK3-treated mice; Fig 4A and 4B). Since MLA (1 nM) decreased basal DA release (p = 0.0210 vs. saline-treated mice; Fig 4A and 4B), α7 nAChR likely mediates basal DA release in the hippocampal CA1 region. DhβE (100 μM), but not MLA (1 nM), significantly blocked SAK3-promoted 5-HT release (DhβE: p > 0.9999 vs. saline-treated mice, p = 0.0314 vs. SAK3-treated mice; MLA: p = 0.0212 vs. saline-treated mice, p > 0.9999 vs. SAK3-treated mice; Fig 4C and 4D). DhβE (100 μM) and MLA (1 nM) did not change basal 5-HT release in the hippocampus (DhβE: p > 0.9999 vs. saline-treated mice; MLA: p = 0.7002 vs. saline-treated mice; Fig 4C and 4D). Two-way repeated measures ANOVA analysis revealed several significant effect on DA (SAK3 + NNC 55–0396 treatment [F (1, 13) = 0.509, p = 0.4880], time [F (15, 195) = 9.946, p < 0.0001] and time × SAK3 + NNC 55–0396 treatment interaction [F (15, 195) = 1.783, p = 0.0393]; SAK3 + DhβE treatment [F (1, 14) = 0.073, p = 0.7907], time [F (15, 210) = 3.949, p < 0.0001] and time × SAK3 + DhβE treatment interaction [F (14, 224) = 0.520, p = 0.9282]; SAK3 + MLA treatment [F (1, 13) = 4.369, p = 0.0568], time [F (15, 195) = 12.802, p < 0.0001] and time × SAK3 + MLA treatment interaction [F (15, 195) = 3.047, p = 0.0002]; SAK3 + TTA-2A treatment [F (1, 14) = 2.232, p = 0.1574], time [F (15, 210) = 3.815, p < 0.0001] and time × SAK3 + TTA-2A treatment interaction [F (15, 210) = 1.148, p = 0.3154]; Fig 4A) and 5-HT (SAK3 + NNC 55–0396 treatment [F (1, 13) = 0.636, p = 0.4393], time [F (15, 195) = 3.837, p < 0.0001] and time × SAK3 + NNC 55–0396 treatment interaction [F (15, 195) = 0.440, p = 0.9654]; SAK3 + DhβE treatment [F (1, 14) = 1.503, p = 0.2404], time [F (15, 210) = 5.681, p < 0.0001] and time × SAK3 + DhβE treatment interaction [F (15, 210) = 0.440, p = 0.9655]; SAK3 + MLA treatment [F (1, 13) = 9.694, p = 0.082], time [F (15, 195) = 4.720, p < 0.0001] and time × SAK3 + MLA treatment interaction [F (15, 195) = 3.990, p < 0.0001]; SAK3 + TTA-2A treatment [F (1, 14) = 41.942, p < 0.0001], time [F (15, 210) = 4.785, p < 0.0001] and time × SAK3 + TTA-2A treatment interaction [F (15, 210) = 4.623, p < 0.0001]; Fig 4C). Taken together, SAK3 promotes DA and 5-HT releases by enhancing T-type calcium channels and activating nAChR in the hippocampal CA1 region.

### Peak response of DA and 5-HT release followed by SAK3 administration is delayed in Cav3.1 KO hippocampal CA1 region

To reveal mechanism underlying SAK3-facilitated hippocampal DA and 5-HT releases, we assessed whether Cav3.1 gene deletion inhibits effects of SAK3 (Fig 5). SAK3 significantly promoted hippocampal DA but not 5-HT releases in Cav3.1 KO mice (DA: p = 0.00485 vs. saline-treated Cav3.1 KO mice; 5-HT: p = 0.278388 vs. saline-treated Cav3.1 KO mice; Fig 4B and 4D), suggesting that SAK3 effects are partially blocked by Cav3.1 gene deletion. Additionally, delayed elevation of DA and 5-HT release followed by SAK3 (0.5 mg/kg, p.o.) administration were observed in Cav3.1 KO hippocampus (Fig 5A and 5C). Two-way repeated measures ANOVA analysis revealed several significant effect on DA (SAK3 + Cav3.1 gene deletion [F (1, 6) = 11.967, p = 0.0135], time [F (15, 90) = 1.736, p = 0.0576] and time × SAK3 + Cav3.1 gene deletion interaction [F (15, 90) = 1.592, p = 0.0917]; Fig 5A) and 5-HT (SAK3 + Cav3.1 gene deletion [F (1, 6) = 0.916, p = 0.3754], time [F (15, 90) = 2.233, p = 0.0104] and time × SAK3 + Cav3.1 gene deletion interaction [F (15, 90) = 1.528, p = 0.1119]; Fig 5C).

### Acute SAK3 administration enhances DA and 5-HT releases in the hippocampal CA1 region in APP^NL-GF^ KI mice

**Fig 6:**
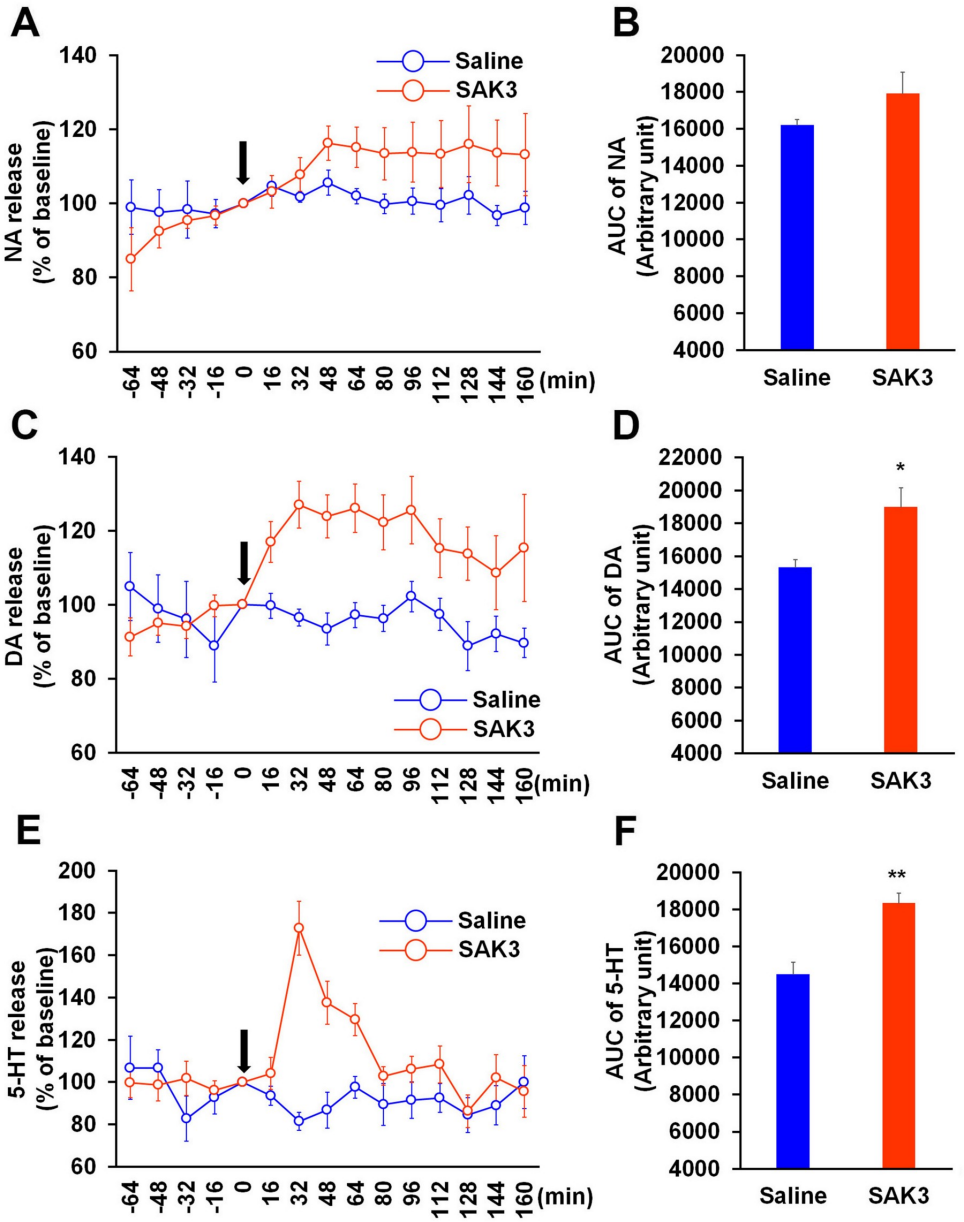
Effects of SAK3 on monoamine releases in the hippocampus of APP^NL-GF^ KI mice. (A, C, E) Time zero indicates the point immediately after SAK3 oral administration. NA, DA, and 5-HT levels in the dialysate were analyzed every 16 min in the hippocampal CA1 region of APP^NL-GF^ KI mice (n = 7 per group). Error bars represent the SEM. (B, D, F) NA, DA, and 5-HT levels following SAK3 (0.5 mg/kg, p.o.) administration were calculated with the AUC (n = 7 per group). **p < 0.01 vs. saline-treated mice. Error bars represent the SEM. APP: amyloid precursor protein, NA: noradrenaline, DA: dopamine, 5-HT: serotonin, AUC: area under the receiver operator characteristic curve.

Finally, we tested whether SAK3 can promote monoamine release in the hippocampus in an animal model of AD. For the analysis, we used 10-month-old APP^NL-GF^ KI mice exhibiting memory impairments due to amyloid plaque formation in the hippocampus [20, 28]. Importantly, SAK3 (0.5 mg/kg, p.o.) administration significantly elevated DA and 5-HT releases in the hippocampus of APP^NL-GF^ KI mice (DA: p = 0.0136 vs. saline-treated mice; 5-HT: p = 0.0006 vs. saline-treated mice; Fig 6D and 6F). SAK3 did not affect NA release in APP^NL-GF^ KI mice (p = 0.1799 vs. saline-treated mice; Fig 6B). Two-way repeated measures ANOVA analysis revealed several significant effect on NA (SAK3 administration [F (1, 12) = 1.218, p = 0.2914], time [F (14, 168) = 2.734, p = 0.0012] and time × SAK3 administration interaction [F (14, 168) = 1.84, p = 0.0365]; Fig 6A), DA (SAK3 administration [F (1, 12) = 6.415, p = 0.0263], time [F (14, 168) = 2.396, p = 0.0045] and time × SAK3 administration interaction [F (14, 168) = 2.858, p = 0.0007]; Fig 6C) and 5-HT (SAK3 administration [F (1, 12) = 11.19, p = 0.0058], time [F (14, 168) = 2.614, p = 0.0019] and time × SAK3 administration interaction [F (14, 168) = 4.314, p < 0.0001]; Fig 6E). We also measured basal monoamine contents in the hippocampal CA1 region and the mPFC of APP^NL-GF^ KI mice. Whereas no differences were observed in monoamine content in the mPFC (NA: p = 0.7913 vs. WT mice; DA: p = 0.1159 vs. WT mice; 5-HT: p = 0.5970 vs. WT mice; Table 2), DA and 5-HT contents were markedly reduced in the hippocampal CA1 region of APP^NL-GF^ KI mice than of WT mice of the same age (NA: p = 0.4602 vs. WT mice; DA: p = 0.0487 vs. WT mice; 5-HT: p = 0.0394 vs. WT mice; Table 2). Therefore, these results indicated that SAK3 can promote hippocampal DA and 5-HT releases under the AD-like condition.

**Table 2:** Monoamine contents in the mPFC and hippocampal CA1 region in APP^NL-GF^ KI mice.

**mPFC**	**Saline**	**SAK3**
NA (ng/g)	523.4 ± 8.5	546.7 ± 37.9
DA (ng/g)	603.5 ± 266.0	179.6 ± 37.5
5-HT (ng/g)	526.7 ± 77.2	485.1 ± 27.4
**Hippocampus**	**Saline**	**SAK3**
NA (ng/g)	513.0 ± 111.6	433.2 ± 21.8
DA (ng/g)	235.1 ± 86.8	52.7 ± 15.0*
5-HT (ng/g)	876.3 ± 95.3	624.1 ± 54.0*

Ten-month-old APP^NL-GF^ KI mice showed reduction in DA and 5-HT contens compared to WT mice of the same age (WT: n = 5; APP^NL-GF^ KI: n = 6) * p < 0.05 vs. WT mice.

mPFC: medial prefrontal cortex, NA: noradrenaline, DA: dopamine, 5-HT: serotonin, WT: wildtype, KI: knock in; APP: amyloid precursor protein

## Discussion

In the present study, we demonstrated that SAK3 promotes DA and 5-HT releases in the naive mouse hippocampus but not in the mPFC. In addition, we also made the following novel observations: (1) T-type calcium channel inhibitor, NNC 55–0396, antagonized SAK3-induced DA and 5-HT releases in the hippocampal CA1 region; (2) α4 nAChR antagonist, DhβE, and/or α7 nAChR antagonist, MLA, blocked DA and 5-HT releases by SAK3 administration in the hippocampus; (3) SAK3 significantly promoted DA and 5-HT releases in the hippocampal CA1 region of APP^NL-GF^ KI mice.

Previous reports have indicated that T-type calcium channel activities play a key role in DA release [29, 30]. Blockade of T-type calcium channels by Ni^2+^ (100 μM) decrease single pulse-evoked DA release in the Hartley guinea pig striatum [29]. High K^+^ levels (60 mM) increase the frequency of DA release in rat dopaminergic neurons in the substantia nigra pars compacta using carbon fiber microelectrodes, and this action is abolished by T-type calcium channel blocker, mibefradil (10 mM) [30]. On the other hand, several voltage-gated calcium channels are involved in 5-HT release. P/Q-type calcium channel inhibitor, ω-agatoxin IVA (0.1–1 μM) and N-type calcium channel inhibitor, ω-conotoxin GVIA (3–10 μM), but not the L-type calcium channel inhibitor, nifedipine (3 μM) antagonized high K^+^ (50 mM)-induced 5-HT release in rat hippocampal slices [31]. We demonstrated here that SAK3 promotes DA and 5-HT releases in the hippocampus, an effect blocked by NNC 55–0396 (1 μM) administration. We previously defined that polymethoxyflavone nobiletin enhances hippocampal DA release via enhancing the T-type calcium channel in naive and 1-methyl-4-phenyl-1,2,3,6-tetrahydropyridine-treated mice [24]. Moreover, an *in situ* hybridization study indicated that T-type calcium channel mRNAs expressed in the rat substantia nigra and raphe nuclei [16], which are the origin of dopaminergic and serotonergic neurons respectively, and T-type calcium channels likely mediate each neuronal activity and development [32–35]. These observations suggest that SAK3 promotes hippocampal DA and 5-HT releases via enhancing T-type calcium channels.

On the other hand, the cholinergic system is known to mediate DA and 5-HT releases in the brain. For example, nicotine (0.3 mg/kg, s.c.) treatment increased DA release in the rat hippocampus, an effect blocked by the α7 nAChR selective antagonist, MLA (500 μM) and non-specific nAChR antagonist, mecamylamine (MEC: 100 μM) used in microdialysis analysis [36]. Administration of low dose nicotine (1 μM) also enhanced hippocampal DA release in rats [37]. Treatment with α7 nAChR agonist, EVP-6124 (0.1 mg/kg, i.p.) also increased DA release in the rat mPFC and nucleus accumbens (NAc) [38]. Electrostimulation-evoked DA release was inhibited by nAChRs inhibitor, hexamethonium (200 μM) administration in the mouse NAc [39]. Likewise, nicotine (50–500 μM) treatment promoted [^3^H]-5-HT release in rat hippocampal slices in a concentration-dependent manner, and MEC (0.5 μM) treatment significantly antagonized it [40]. On the other hand, nicotine treatment decreases 5-HT release in the rat hippocampus using *in vivo* microdialysis [36, 41]. Since nicotine stimulation promoted both monoamine and gamma-aminobutyric acid (GABA) release [42], GABA inhibitory neurotransmission may contribute to these discrepancies between *in vivo* and *ex vivo* conditions. Furthermore, nicotine stimulation increased the frequency of excitatory postsynaptic currents in serotonergic neurons in the dorsal raphe nucleus through the α4β2 nAChRs [43], suggesting that the nAChR may mediate serotonergic neuronal activity. Previously, we indicated that SAK3 promoted ACh release in the mouse hippocampus, an effect inhibited by T-type calcium channel inhibitor treatment and/or by deficiency of the *Cav3*.*1* gene [18]. Moreover, SAK3 activates nAChR signaling in hippocampal CA1 pyramidal neurons [18]. Therefore, SAK3 possibly enhances DA and 5-HT releases in the hippocampus via indirect stimulation of nAChR. However, SAK3 could not alter DA and 5-HT releases in the mPFC. Whereas cholinergic innervation in the mPFC is received from the nucleus basalis of Meynert, where it may show low expression levels of T-type calcium channel mRNAs [16, 44], cholinergic neurons in medial septum input to the hippocampus [44]. Since T-type calcium channels are highly expressed in the medial septum [16, 19], the fact that SAK3 did not affect the monoamines in the mPFC may be due to differences in cholinergic innervation. Further studies are required to define the action mechanism of SAK3 on DA and 5-HT releases in the brain.

Previous studies have indicated that NA release is mediated only by N-type calcium channels and not by any other type of voltage-gated calcium channels [45–47]. In rat hippocampal slices, nAChR agonist dimethylphenylpiperazinium-induced [^3^H]-NA release is significantly blocked by N-type calcium channel inhibitors and not by other voltage-gated calcium channel blockers [47]. By contrast, functional L-type and T-type calcium channels are expressed and regulate neuronal pacemaking in the locus ceruleus located in noradrenergic neurons [48]. While the combined application of the L-type calcium channel inhibitor, isradipine (120 nM) and the T-type calcium channel inhibitor, mibefradil (2 μM) increases firing frequency and decreases afterhyperpolarization amplitude in noradrenergic neurons, mibefradil (2 μM) alone could not affect pacemaking, suggesting that both channel functions may be essential for neuronal activity in the locus ceruleus [48]. Thus, we concluded here that the T-type calcium channel enhancer SAK3 could not affect NA release in the mouse brain.

Several postmortem studies have reported decreased density of dopamine receptors in the AD brain including the hippocampus [49–51]. In addition, D1-like receptor agonists significantly improve memory deficits seen in amyloid β-injected mice [52, 53]. On the other hand, 5-HT concentration was significantly decreased in the platelets in patients with AD [54] and selective serotonin reuptake inhibitors improve decreased cognitive performance and BPSD seen in patients with AD [55, 56]. Likewise, in the AD brain, reduction of DA and 5-HT contents was observed in the hippocampus of APP^NL-GF^ KI mice, suggesting that decrease in dopaminergic and serotonergic pathways in the hippocampus may be associated with the cognitive impairments seen in APP^NL-GF^ KI mice [20]. In addition, SAK3 could enhance DA and 5-HT release in the hippocampus in APP^NL-GF^ KI mice. These observations suggest that SAK3 may have potential for improvement of cognitive impairments seen in APP^NL-GF^ KI mice. Supported this idea, we reported that chronic SAK3 (0.5 mg/kg, p.o.) administration significantly antagonizes cognitive impairments seen in APP^NL-F^ KI mice [22]. Therefore, SAK3 may be able to become an attractive therapeutic for both cognitive impairment and BPSD observed in patients with AD.

Here, we observed that SAK3-promoted DA and 5-HT releases reach a peak at 12 min in the hippocampal CA1 region. We previously reported that the brain concentration of SAK3 reaches approximately 0.2 nM within 15 min after SAK3 (0.5 mg/kg, p.o.) administration [22]. Since patch-clamp experiments indicate that 0.1 nM SAK3 can enhance Cav3.1 and 3.3 currents maximally in Cav3.1 and Cav3.3 over-expressed neuro2A cells [18], SAK3 (0.5 mg/kg, p.o.) administration in the present study may be enough to act in the brain.

While T-type calcium channel inhibitor NNC 55–0396 (1 μM) significantly inhibited SAK3-promoted hippocampal DA and 5-HT releases, the same dose of TTA-A2 (1 μM) failed to inhibit SAK3 effect completely. Previous reports indicated that IC_50_ of TTA-A2 on Cav3.1 T-type calcium channel (89 nM) is stronger than that of NNC 55–0396 (6.8 μM) by in vitro assay [23, 57]. Since these experiments were done by different conditions such as holding potential (NNC 55–0396: -70 mV; TTA-A2: -80 mV), external and internal solutions [23, 57], it may be difficult to compare the inhibition potentials between NNC 55–0396 and TTA-A2. Moreover, TTA-A2 effect on T-type calcium channels in vivo is still unknown [57, 58]. As NNC 55–0396 (1 μM) application significantly blocks SAK3 (0.5 mg/kg, p.o.)-promoted hippocampal ACh release [18], we here concluded that TTA-A2 (1 μM) is not enough to suppress T-type calcium channel activity using *in vivo* microdialysis. Cav3.1 gene deletion also partially inhibited the SAK3 effect, suggesting that Cav3.1 in part mediates the initiation of hippocampal DA and 5-HT release. We have reported that SAK3 enhances Cav3.1 and 3.3 currents, and that SAK3-promoted hippocampal ACh release is partially attenuated in Cav3.1 KO mice [18]. Thus, Cav3.3 activity may in part mediate SAK3 effects on DA and 5-HT releases in Cav3.1 KO hippocampus. Further studies are required to define the mechanism underlying SAK3-promoted monoamine release in the mouse hippocampus.

In summary, the T-type calcium channel enhancer SAK3 promoted DA and 5-HT, but not NA, release in the mouse hippocampus under naive and AD-like conditions. SAK3 may promote DA and 5-HT releases through activation of the nAChR by enhancing ACh release in the hippocampus. Therefore, SAK3 can facilitate both ACh and DA and 5-HT releases in the hippocampus through enhancing T-type calcium channels. This evidence is particularly important for SAK3-induced improvement of memory deficits and BPSD seen in patients with AD.

## Supporting information

S1 File Low data. (XLSX)

## Abbreviations

5-HT: serotonin
ACh: Acetylcholine
AD: Alzheimer’s disease
ANOVA: analysis of variance
APP: amyloid precursor protein
AUC: area under the curve
BPSD: behavioral and psychological symptoms of dementia
DA: dopamine
DhβE: dihydro-β-erythroidine
ECD: electrochemical detector
GABA: gamma-aminobutyric acid
HPLC: high-performance liquid chromatography
KI: knock-in
KO: knock-out
MEC: mecamylamine
MLA: methyllycaconitine
mPFC: medial prefrontal cortex
NA: noradrenaline
NAc: nucleus accumbens
nAChR: nicotinic acetylcholine receptor
SAK3: ethyl 8'-methyl-2',4-dioxo-2-(piperidin-1-yl)-2'H-spiro[cyclopentane-1,3'-imidazo [1,2-a] pyridine]-2-ene-3-carboxylate
ST101: spiro [imidazole [1.2-a] pyridine-3, 2-indan]-2(3H)-one
WT: wild type
