# Correction: T-type calcium channel enhancer SAK3 promotes dopamine and serotonin releases in the hippocampus in naive and amyloid precursor protein knock-in mice

**DOI:** 10.1371/journal.pone.0211590

**Published:** 2019-01-25

**Authors:** Shuo Wang, Yasushi Yabuki, Kazuya Matsuo, Jing Xu, Hisanao Izumi, Kenji Sakimura, Takashi Saito, Takaomi C. Saido, Kohji Fukunaga

In the Funding section, one of the grant numbers from the funder Scientific Research from the Ministry of Education, Science, Sports, and Culture of Japan is missing. The correct grant numbers are: Kakenhi 25293124 to K.F., 17K15456 to Y.Y. and 18J20651 to K.M.
