# Expression of Concern: T-type calcium channel enhancer SAK3 promotes dopamine and serotonin releases in the hippocampus in naive and amyloid precursor protein knock-in mice

**DOI:** 10.1371/journal.pone.0280120

**Published:** 2023-01-11

**Authors:** The PLOS ONE Editors

The Funding Statement for this article [1, 2] states that the study received funding from the Smoking Research Foundation, which according to [3] has received financial support from the tobacco industry. In light of this issue the *PLOS ONE* article [1, 2] does not comply with the journal’s policy on Funding from Tobacco Companies [4] which was implemented in 2010.

Therefore, the *PLOS ONE* Editors issue this Expression of Concern.

We regret that this concern was not identified and addressed prior to the article’s [1, 2] publication.
